# Physical Activity During the Coronavirus (COVID-19) Pandemic: Prevention of a Decline in Metabolic and Immunological Functions

**DOI:** 10.3389/fspor.2020.00057

**Published:** 2020-04-30

**Authors:** Johan Jakobsson, Christer Malm, Maria Furberg, Ulf Ekelund, Michael Svensson

**Affiliations:** ^1^Section of Sports Medicine, Department of Community Medicine and Rehabilitation, Umeå University, Umeå, Sweden; ^2^Department of Clinical Microbiology, Umeå University, Umeå, Sweden; ^3^Department of Sports Medicine, Norwegian School of Sport Sciences, Oslo, Norway; ^4^Norwegian Institute of Public Health, Oslo, Norway

**Keywords:** physical activity, COVID-19, sedentary behavior, health, mortality, coronavirus, SARS-CoV-2

## Introduction

As of 30th January 2020, the outbreak of the novel coronavirus disease, later called COVID-19, was declared a Public Health Emergency (World Health Organization, 2020b) and on the 11th of March 2020, COVID-19 was characterized as a pandemic (World Health Organization, 2020d). On April 20th 2020, WHO reported 2,314,621 confirmed cases of COVID-19 and a total of 157,847 deaths from all regions of the world (World Health Organization, 2020a).

Governments' immediate protective measurements aim to slow down the ongoing spread of the COVID-19 disease. Restrictions include full or partial lockdowns of cities, travel bans, restricted social gatherings, and closed borders and schools. Half of humanity is currently directly affected by the COVID-19 pandemic (The New York Times, 2020), with uncertain final consequences. Likely, our daily life with physical activity (PA) will be impaired for months, affecting the health of a significant portion of society. A significant decline in steps taken assessed by activity tracker users has already been shown (Fitbit, 2020).

## Importance of Physical Activity

Encouraging or mandating that people should remain within their homes with discontinued daily life activities may unintentionally increase sedentary behavior, decrease general PA, and inflict negative health consequences. Decreased PA will lower mechanical load, metabolic rate, and energy expenditure, which may result in a decline in physical fitness and an energy surplus. All are well-known risk-factors for future disease manifestations, imposing further economic burden on tomorrow's society (Owen et al., 2010; Malm et al., 2019).

What was termed the global pandemic of physical inactivity in 2012 was estimated to be the cause of 5.3 million deaths per year (Das and Horton, 2016). A sedentary behavior with high levels of sitting time and low levels of PA are associated with increased risks of depression (Huang et al., 2020), type 2 diabetes, cancer (Patterson et al., 2018), coronary vascular disease (CVD), and mortality (Stamatakis et al., 2019). The human physiology responds quickly to reduced PA. For example, two weeks of reduced PA leads to a decrease in cardiorespiratory fitness and multi-organ insulin sensitivity (Bowden Davies et al., 2018). Just 1 week of induced sedentary behavior has deleterious effects on mood and depression (Edwards and Loprinzi, 2016). Also, 1 week of reduced step count by 91% significantly reduced myofibrillar protein synthesis rates and upregulated muscle atrophy in male adults (Shad et al., 2019). Further, immobilization and sedentary behavior, such as TV viewing, are strong risk factors for venous thromboembolism (Kubota et al., 2018). On the contrary, regular PA and low sedentary time are associated with reduced risk for morbidity and all-cause mortality (Ekelund et al., 2019).

Early reports on COVID-19 show that individuals of an older age with multiple comorbidities are more prone to developing severe complications following infection by COVID-19 and have an increased risk of mortality (Emami et al., 2020). These observations indirectly indicate that low fitness, obesity, and an altered immune system could be detrimental for an individual exposed to the coronavirus, SARS-CoV-2. The effects of COVID-19 on patients with obesity is yet to be well-described, however, several reports identified obesity as a risk factor for hospitalization (Dietz and Santos-Burgoa, 2020).

Severe detrimental effects of COVID-19 include lung damage, pneumonia, an overwhelming innate-induced inflammation (Liu et al., 2020), abnormal coagulation characteristics (Tang et al., 2020), and cardiac and kidney injury (Chen et al., 2020). In this context, documented preventive and alleviating effects of PA on complications by viral infections are of significant interest. Firstly, regular PA improves cardiovascular functions (Pinckard et al., 2019), coagulation and fibrinolytic homeostasis (Lippi and Maffulli, 2009), and overall protection to cellular stress (Narasimhan and Rajasekaran, 2016). Secondly, PA can increase the endurance and strength of the respiratory muscles, making them more efficient (McKenzie, 2012). Thirdly, regular PA has positive effects on the immune system (Dorneles et al., 2020), may restrain the immunosenescence generally seen with aging (Weyh et al., 2020), and can enhance the immune response to viral antigens, lowering the incidence of viral infections across the lifespan (Campbell and Turner, 2018).

Particularly, PA during aging may have positive effects on the adaptive immune system including T and B lymphocytes (Turner, 2016). For instance, interleukin-15 (IL-15), a pleiotropic cytokine crucial for the activation and proliferation of T and NK cells which is highly expressed in muscle fibers, is released following exercise (Nielsen et al., 2007). An exercise-induced release of IL-15 from skeletal muscles may provide positive effects to the immune system, as one of many health-preserving effects caused by exercise. Regular PA may also have positive effects on the immune-system via other mechanisms, such as the prevention of a low-grade inflammation generally seen in central obesity (Collao et al., 2020) which is strongly associated with an increased risk of type 2 diabetes and CVD. Collectively, PA and prevention of obesity are crucial in the prevention of severe complications and premature mortality in future viral pandemics.

Physical activity also has a major role in mental health and cognitive function because exercise has positive effects in preventing and alleviating depressive symptoms (Schuch et al., 2016), lessening anxiety (Stubbs et al., 2017), improving learning (Winter et al., 2007), and is beneficial for cognitive functioning in older adults (Bangsbo et al., 2019). In addition to promoting PA, taking part in sports gives the participant a chance for psychosocial development, being part of a community, and developing a social network (Holt et al., 2017). With limited social activities due to mandated restrictions, organized sporting activities will vastly decrease during the virus outbreak. Consequently, continued PA is invaluable for maintaining good physical and mental health when tackling current challenges imposed upon us by COVID-19.

## Attenuate Negative Health Consequences

Physical exercise at home is easily carried out and will help maintain fitness levels. Also, performing household tasks contributes significantly to total energy expenditure, especially for the elderly (Ratzlaff et al., 2010). The World Health Organization exemplifies some practical home-based exercises, available at their website, including: squats, bridges, back extensions, and chair dips, to be performed for 10–15 repetitions up to five times with 1-min rest between sets (World Health Organization, 2020c). Further, planks and superman can be performed for strengthening the core, while knee to elbow and side knee lifts, to name a few, can be used for increasing the heart rate and respiratory rate.

Even with the restrictions of limited space or lack of special equipment, reaching the WHO recommendations of 150 min of moderate-intensity or 75 min of vigorous-intensity PA per week (World Health Organization, 2010; Piercy and Troiano, 2018) is still achievable even at home during self-isolation. For additional health benefits, 300 min of moderate-intensity or 150 min of vigorous-intensity PA per week is recommended. In the case of dramatically decreased PA due to mandated restrictions, and thus, vastly decreased non-exercise activity thermogenesis, reaching the higher end of the recommendations could be valuable. A combination of muscle-strengthening exercises for the major muscle groups, walking, stair climbing, and performing household tasks, is recommended to maintain PA during the coronavirus crisis. Exercising outside is, of course, an option, while maintaining a distance from other people. While moderate PA is beneficial for health and immune function, the risk of illness may be elevated during periods of unusually heavy exertion (Simpson et al., 2020). Accordingly, follow the guidelines from WHO or your public health agency.

It is also important to interrupt prolonged sitting time, as this is beneficial for metabolic health, i.e., insulin sensitivity and blood lipids (Loh et al., 2020). For instance, regular interruptions of 3-min of light-intensity activities during 210 min of sedentary time can diminish the glycemic response following a high-energy meal, valuable for glycemic control (Climie et al., 2018). Homestay and working from home likely increase sitting time, specifically screen time in front of the TV, mobile phone, and computer. Regular interruptions are therefore suggested.

To increase motivation for PA, internet-delivered interventions through the computer or mobile phone, or self-monitoring through diaries or phone apps, are viable tools for exercise motivation (Tate et al., 2015). Also, one can follow an online exercise class, often freely available on different media platforms. While exercise is safe for most individuals, the elderly and those who are susceptible to cardiovascular or other complications should consult health providers before starting a new exercise regime.

## Conclusions

Maintaining regular PA during self-isolation is important for the prevention of future chronic health conditions due to a sedentary lifestyle. During crises, functional medical care and vital societal services are of the highest priority. To prevent additional physical and mental distress, governments, public health authorities, and the public itself should care also for maintaining PA during the COVID-19 pandemic. In summary:

- All PA is beneficial and doing something is better than doing nothing
- Interrupt prolonged sitting time and reduce sedentariness with short active breaks during the day
- Accumulate at least 150 min of moderate-intensity or 75 min of vigorous-intensity PA per week
- When desired, use a training app for the monitoring of activity and/or follow an online exercise class for exercise motivation
- Include both muscle-strengthening and cardiovascular exercises
- Always be cautious and aware of your own limitations, and never do exercise with an ongoing infection.

Additionally, regular PA is important in the prevention of severe complications in any future pandemic viruses similar to COVID-19.

## Author Contributions

JJ conceptualized the paper and wrote the first edition of the manuscript. JJ, CM, MF, UE, and MS contributed to the manuscript with their expertise, read, edited, and approved the submitted version.

## Conflict of Interest

The authors declare that the research was conducted in the absence of any commercial or financial relationships that could be construed as a potential conflict of interest.

## References

[B1] Bangsbo J. Blackwell J. Boraxbekk C.-J. Caserotti P. Dela F. Evans A. B.. (2019). Copenhagen consensus statement 2019: physical activity and ageing. Br. J. Sports Med. 53:856. 10.1136/bjsports-2018-100451 30792257 PMC6613739

[B2] Bowden Davies K. A. Sprung V. S. Norman J. A. Thompson A. Mitchell K. L. Halford J. C. G.. (2018). Short-term decreased physical activity with increased sedentary behaviour causes metabolic derangements and altered body composition: effects in individuals with and without a first-degree relative with type 2 diabetes. Diabetologia 61, 1282–1294. 10.1007/s00125-018-4603-5 29671031

[B3] Campbell J. P. Turner J. E. (2018). Debunking the myth of exercise-induced immune suppression: redefining the impact of exercise on immunological health across the lifespan. Front. Immunol. 9:648. 10.3389/fimmu.2018.00648 29713319 PMC5911985

[B4] Chen T. Wu D. Chen H. Yan W. Yang D. Chen G.. (2020). Clinical characteristics of 113 deceased patients with coronavirus disease 2019: retrospective study. BMJ. 368:m1091. 10.1136/bmj.m1091 32217556 PMC7190011

[B5] Climie R. E. Grace M. S. Larsen R. L. Dempsey P. C. Oberoi J. Cohen N. D.. (2018). Regular brief interruptions to sitting after a high-energy evening meal attenuate glycemic excursions in overweight/obese adults. Nutr. Metab. Cardiovasc. Dis. 28, 909–916. 10.1016/j.numecd.2018.05.009 30111495

[B6] Collao N. Rada I. Francaux M. Deldicque L. Zbinden-Foncea H. (2020). Anti-inflammatory effect of exercise mediated by Toll-like receptor regulation in innate immune cells - a review. Int. Rev. Immunol. 39, 39–52. 10.1080/08830185.2019.1682569 31682154

[B7] Das P. Horton R. (2016). Physical activity-time to take it seriously and regularly. Lancet 388, 1254–1255. 10.1016/S0140-6736(16)31070-4 27475269

[B8] Dietz W. Santos-Burgoa C. (2020). Obesity and its implications for COVID-19 mortality. Obesity. 10.1002/oby.22818. [Epub ahead of print]. 32237206

[B9] Dorneles G. P. Dos Passos A. A. Z. Romao P. R. T. Peres A. (2020). New insights about regulatory T cells distribution and function with exercise: the role of immunometabolism. Curr Pharm Des. 26, 979–990. 10.2174/1381612826666200305125210 32133958

[B10] Edwards M. K. Loprinzi P. D. (2016). Effects of a sedentary behavior-inducing randomized controlled intervention on depression and mood profile in active young adults. Mayo Clin. Proc. 91, 984–998. 10.1016/j.mayocp.2016.03.021 27492908

[B11] Ekelund U. Tarp J. Steene-Johannessen J. Hansen B. H. Jefferis B. Fagerland M. W.. (2019). Dose-response associations between accelerometry measured physical activity and sedentary time and all cause mortality: systematic review and harmonised meta-analysis. BMJ 366:l4570. 10.1136/bmj.l4570 31434697 PMC6699591

[B12] Emami A. Javanmardi F. Pirbonyeh N. Akbari A. (2020). Prevalence of underlying diseases in hospitalized patients with COVID-19: a systematic review and meta-analysis. Arch. Acad. Emerg. Med. 8:e35. 10.22037/aaem.v8i1.600 32232218 PMC7096724

[B13] Fitbit (2020). The Impact Of Coronavirus On Global Activity. Available online at: https://blog.fitbit.com/covid-19-global-activity/ (accessed April 5, 2020).

[B14] Holt N. L. Neely K. C. Slater L. G. Camire M. Cote J. Fraser-Thomas J.. (2017). A grounded theory of positive youth development through sport based on results from a qualitative meta-study. Int. Rev. Sport Exerc. Psychol. 10, 1–49. 10.1080/1750984X.2016.1180704 27695511 PMC5020349

[B15] Huang Y. Li L. Gan Y. Wang C. Jiang H. Cao S.. (2020). Sedentary behaviors and risk of depression: a meta-analysis of prospective studies. Transl. Psychiatry 10, 26–26. 10.1038/s41398-020-0715-z 32066686 PMC7026102

[B16] Kubota Y. Cushman M. Zakai N. Rosamond W. D. Folsom A. R. (2018). TV viewing and incident venous thromboembolism: the Atherosclerotic Risk in Communities Study. J. Thromb. Thrombolysis 45, 353–359. 10.1007/s11239-018-1620-7 29468488 PMC6231408

[B17] Lippi G. Maffulli N. (2009). Biological influence of physical exercise on hemostasis. Semin. Thromb. Hemost. 35, 269–276. 10.1055/s-0029-1222605 19452402

[B18] Liu J. Zheng X. Tong Q. Li W. Wang B. Sutter K.. (2020). Overlapping and discrete aspects of the pathology and pathogenesis of the emerging human pathogenic coronaviruses SARS-CoV, MERS-CoV, and 2019-nCoV. J. Med. Virol. 92, 491–494. 10.1002/jmv.25709 32056249 PMC7166760

[B19] Loh R. Stamatakis E. Folkerts D. Allgrove J. E. Moir H. J. (2020). Effects of interrupting prolonged sitting with physical activity breaks on blood glucose, insulin and triacylglycerol measures: a systematic review and meta-analysis. Sports Med. 50, 295–330. 10.1007/s40279-019-01183-w 31552570 PMC6985064

[B20] Malm C. Jakobsson J. Isaksson A. (2019). Physical activity and sports-real health benefits: a review with insight into the public health of Sweden. Sports 7:127. 10.3390/sports7050127 31126126 PMC6572041

[B21] McKenzie D. C. (2012). Respiratory physiology: adaptations to high-level exercise. Br. J. Sports Med. 46:381. 10.1136/bjsports-2011-090824 22267571

[B22] Narasimhan M. Rajasekaran N. S. (2016). Exercise, Nrf2 and antioxidant signaling in cardiac aging. Front. Physiol. 7:241. 10.3389/fphys.2016.00241 27378947 PMC4911351

[B23] Nielsen A. R. Mounier R. Plomgaard P. Mortensen O. H. Penkowa M. Speerschneider T.. (2007). Expression of interleukin-15 in human skeletal muscle effect of exercise and muscle fibre type composition. J. Physiol. 584(Pt 1), 305–312. 10.1113/jphysiol.2007.139618 17690139 PMC2277063

[B24] Owen N. Sparling P. B. Healy G. N. Dunstan D. W. Matthews C. E. (2010). Sedentary behavior: emerging evidence for a new health risk. Mayo Clin. Proc. 85, 1138–1141. 10.4065/mcp.2010.0444 21123641 PMC2996155

[B25] Patterson R. McNamara E. Tainio M. de Sa T. H. Smith A. D. Sharp S. J.. (2018). Sedentary behaviour and risk of all-cause, cardiovascular and cancer mortality, and incident type 2 diabetes: a systematic review and dose response meta-analysis. Eur. J. Epidemiol. 33, 811–829. 10.1007/s10654-018-0380-1 29589226 PMC6133005

[B26] Piercy K. L. Troiano R. P. (2018). Physical activity guidelines for Americans from the US Department of Health and Human Services. Circ. Cardiovasc. Qual. Outcomes 11:e005263. 10.1161/CIRCOUTCOMES.118.005263 30571339

[B27] Pinckard K. Baskin K. K. Stanford K. I. (2019). Effects of exercise to improve cardiovascular health. Front. Cardiovsc. Med. 6:69. 10.3389/fcvm.2019.00069 31214598 PMC6557987

[B28] Ratzlaff C. R. Doerfling P. Steininger G. Koehoorn M. Cibere J. Liang M.. (2010). Lifetime trajectory of physical activity according to energy expenditure and joint force. Arthritis Care Res. 62, 1452–1459. 10.1002/acr.20243 20506184

[B29] Schuch F. B. Vancampfort D. Richards J. Rosenbaum S. Ward P. B. Stubbs B. (2016). Exercise as a treatment for depression: a meta-analysis adjusting for publication bias. J. Pshyciatrc. Res. 77, 42–51. 10.1016/j.jpsychires.2016.02.023 26978184

[B30] Shad B. J. Thompson J. L. Holwerda A. M. Stocks B. Elhassan Y. S. Philp A.. (2019). One week of step reduction lowers myofibrillar protein synthesis rates in young men. Med. Sci. Sports Exerc. 51, 2125–2134. 10.1249/MSS.0000000000002034 31083048

[B31] Simpson R. J. Campbell J. P. Gleeson M. Kruger K. Nieman D. C. Pyne D. B.. (2020). Can exercise affect immune function to increase susceptibility to infection? Exerc. Immunol. Rev. 26, 8–22. 32139352

[B32] Stamatakis E. Gale J. Bauman A. Ekelund U. Hamer M. Ding D. (2019). Sitting time, physical activity, and risk of mortality in adults. J. Am. Coll. Cardiol 73, 2062–2072. 10.1016/j.jacc.2019.02.031 31023430

[B33] Stubbs B. Vancampfort D. Rosenbaum S. Firth J. Cosco T. Veronese N.. (2017). An examination of the anxiolytic effects of exercise for people with anxiety and stress-related disorders: a meta-analysis. Psychiatry Res. 249, 102–108. 10.1016/j.psychres.2016.12.020 28088704

[B34] Tang N. Li D. Wang X. Sun Z. (2020). Abnormal coagulation parameters are associated with poor prognosis in patients with novel coronavirus pneumonia. J. Thromb. Haemost. 18, 844–847. 10.1111/jth.14768 32073213 PMC7166509

[B35] Tate D. F. Lyons E. J. Valle C. G. (2015). High-tech tools for exercise motivation: use and role of technologies such as the internet, mobile applications, social media, and video games. Diabetes Spectr. 28:45. 10.2337/diaspect.28.1.45 25717278 PMC4334081

[B36] The New York Times (2020). Coronavirus news update. The New York Times. Available online at: https://www.nytimes.com/2020/04/03/world/coronavirus-news-updates.html#link-290c3c8 (accessed April 04, 2020).

[B37] Turner J. E. (2016). Is immunosenescence influenced by our lifetime “dose” of exercise? Biogerontology 17, 581–602. 10.1007/s10522-016-9642-z 27023222 PMC4889625

[B38] Weyh C. Kruger K. Strasser B. (2020). Physical activity and diet shape the immune system during aging. Nutrients 12:622. 10.3390/nu12030622 32121049 PMC7146449

[B39] Winter B. Breitenstein C. Mooren F. C. Voelker K. Fobker M. Lechtermann A.. (2007). High impact running improves learning. Neurobiol. Learn Mem. 87, 597–609. 10.1016/j.nlm.2006.11.003 17185007

[B40] World Health Organization (2010). Global Recommendations on Physical Activity for Health. Geneva: World Health Organization. 26180873

[B41] World Health Organization (2020a). Coronavirus Disease 2019 (COVID-19). Situation Report - 91. Available online at: https://www.who.int/docs/default-source/coronaviruse/situation-reports/20200420-sitrep-91-covid-19.pdf?sfvrsn=fcf0670b_4 (accessed April 20, 2020).

[B42] World Health Organization (2020b). Statement on the Second Meeting of the International Health Regulations (2005) Emergency Committee Regarding the Outbreak of Novel Coronavirus (2019-nCoV). Available online at: https://www.who.int/news-room/detail/30-01-2020-statement-on-the-second-meeting-of-the-international-health-regulations-(2005)-emergency-committee-regarding-the-outbreak-of-novel-coronavirus-(2019-ncov) (accessed March 30, 2020).

[B43] World Health Organization (2020c). Stay Physically Active During Self-Quarantine. Available online at: http://www.euro.who.int/en/health-topics/health-emergencies/coronavirus-covid-19/novel-coronavirus-2019-ncov-technical-guidance/stay-physically-active-during-self-quarantine (accessed March 30, 2020).

[B44] World Health Organization (2020d). WHO Director-General's Opening Remarks at the Media Briefing on COVID-19 - 11 March 2020. Available online at: https://www.who.int/dg/speeches/detail/who-director-general-s-opening-remarks-at-the-media-briefing-on-covid-19---11-march-2020 (accessed March 31, 2020).

